# Nested Sampling for Bayesian Model Comparison in the Context of *Salmonella* Disease Dynamics

**DOI:** 10.1371/journal.pone.0082317

**Published:** 2013-12-20

**Authors:** Richard Dybowski, Trevelyan J. McKinley, Pietro Mastroeni, Olivier Restif

**Affiliations:** Department of Veterinary Medicine, University of Cambridge, Cambridge, United Kingdom; Albert Einstein College of Medicine, United States of America

## Abstract

Understanding the mechanisms underlying the observed dynamics of complex biological systems requires the statistical assessment and comparison of multiple alternative models. Although this has traditionally been done using maximum likelihood-based methods such as Akaike's Information Criterion (AIC), Bayesian methods have gained in popularity because they provide more informative output in the form of posterior probability distributions. However, comparison between multiple models in a Bayesian framework is made difficult by the computational cost of numerical integration over large parameter spaces. A new, efficient method for the computation of posterior probabilities has recently been proposed and applied to complex problems from the physical sciences. Here we demonstrate how nested sampling can be used for inference and model comparison in biological sciences. We present a reanalysis of data from experimental infection of mice with *Salmonella enterica* showing the distribution of bacteria in liver cells. In addition to confirming the main finding of the original analysis, which relied on AIC, our approach provides: (a) integration across the parameter space, (b) estimation of the posterior parameter distributions (with visualisations of parameter correlations), and (c) estimation of the posterior predictive distributions for goodness-of-fit assessments of the models. The goodness-of-fit results suggest that alternative mechanistic models and a relaxation of the quasi-stationary assumption should be considered.

## Introduction

### Model comparison

Model-based inference is widely used in life sciences in order to assess the plausibility of hypothesised biological mechanisms based on data from observations or experiments. One of the most common approaches to compare competing models representing alternative hypotheses relies on Akaike's Information Criterion (AIC) [1]. For a given data set 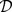, the plausibility of the candidate models 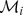 is assessed by calculating their respective AIC values, 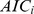:
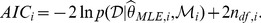
(1)In (1), 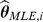 is the maximum likelihood estimate of the set parameters associated with model 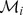, and 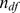 is the corresponding number of degrees of freedom. If 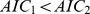 then 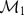 is more plausible than 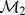, with respect to 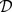, in the sense that the Kullback-Liebler divergence of 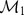 from the true model is smaller [2].

An important drawback to the classic approach to model choice is that it is based on a single point estimate 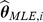 of 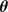, the uncertainty in 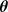 being ignored. In contrast, the Bayesian approach considers a probability distribution for 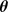, with 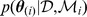 expressing the uncertainty in 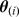 given 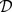 (for a model 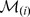).

Suppose that we wish to select a model from a set of candidate models 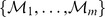 given our observation of data 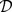. We can express this goal probabilistically by stating that the aim is to determine the most probable model: 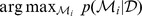.

From Bayes' theorem, we have
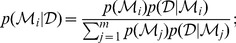
(2)therefore, if 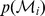 is known, or considered to be equal for all 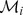 then the focus is on the model evidence 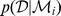.

If 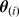 is the set of parameters associated with model 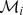, the Bayesian approach to 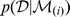 is to integrate over all possible values of 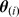:
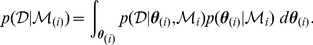
(3)


In addition to allowing for parameter uncertainty, (3) intrinsically penalizes against models that are better able to fit to observed data because of their complexity [3], thereby removing the need for an explicit complexity penalization term.

The integral of (3) can be estimated analytically or numerically. In analytical approaches, the integral is approximated by the adoption of simplifying assumptions; for example, as used for derivation of the Bayes Information Criterion [4]. Numerical approaches are based on some form of Monte Carlo sampling such as Gibbs Sampling [5].

One approach to estimating the integral
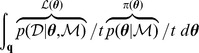
numerically is to sample 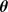 randomly from its prior,
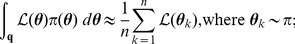
(4)however, the prior 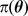 is often concentrated in places where the likelihood 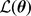 is relatively low. This problem becomes more severe in high-dimensional parameter 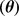 spaces, or in problems where the likelihood function 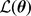 is concentrated in a very small region.

To overcome the problem, Skilling [6], [7] proposed a means of estimating 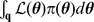 that, by design, samples 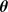 sparsely from the 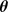 space where the likelihood 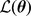 is low, and densely where 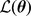 is high, by means of ‘nested sampling’, which is the focus of this paper. A recent addition to the Bayesian arsenal, nested sampling has been used in cosmology to compare alternative models of the universe against observed data [8]. Outside of physics, it has, so far, received little attention [9], [10].

### Within-host dynamics of a bacterial infection

Quantitative research on infectious disease dynamics has undergone rapid development over the last two decades, motivated by concerns about emerging infections that can spread globally and about the evolution of pathogens resistant to existing control measures such as antimicrobials and vaccines. Bayesian computation has become the method of choice to fit stochastic dynamic models to epidemiological [11] or experimental datasets [12]. This is in large part due to the appeal of being able to produce measures of uncertainty and correlation for the model parameters based on their posterior probability distributions. Similarly, models for within-host dynamics of infection have more recently started to benefit from Bayesian inference approaches [13].

*Salmonella enterica* causes systemic diseases (typhoid and paratyphoid fever) [14], food-borne gastroenteritis and non-typhoidal septicaemia (NTS) [15] in humans and in many other animal species world-wide, which also cause a very serious problem for the food industry. The global burden of typhoid fever is estimated at ca. 22 million cases with a mortality estimated at ca. 200,000 deaths per year [14], [16]. Paratyphoid has an estimated 5.4 million illnesses worldwide [16]. The high incidence of these diseases, that affect both travellers to and residents in endemic areas, and threaten infants, children and immunodeficient patients, dictates the urgent need for more efficacious preventive and therapeutic measures.

In the mouse model of systemic infection, *Salmonella* reside and proliferate mainly within phagocytic cells of the spleen liver, bone marrow and lymph nodes [17]–[19]. Observation of *Salmonella* by fluorescence microscopy in the tissues of mice has revealed that a key feature of systemic infections with wild type bacteria is the presence, on average, of low bacterial numbers within individual phagocytes irrespective of net bacterial growth rate and time since infection [20]–[23].

In an effort to understand the dynamics that underpin the intracellular numerical distributions of *Salmonella* within the host cells, and to capture the essential traits of the cell-to-cell spread of the bacteria, we have used mathematical model frameworks for the intensity of intracellular infection that links the quasi-stationary distribution of bacteria to bacterial and cellular demography. An example of this the work done by Brown et al. [24], who compared the observed distribution 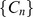, where 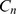 is the number of cells with 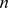 bacteria, across 16 candidate infection models. The models under consideration were as follows: (a) one homogeneous model, in which, for every cell, burst occurred only when the number of bacteria 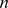 in a cell reached a single burst threshold 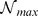; (b) five heterogeneous models having a probability distribution of burst thresholds; and (c) eight stochastic models for which there is a probability that a given cell will undergo burst. Two datasets were analysed, one for a virulent strain of bacteria and the other for an attenuated strain. Brown et al. [24] computed the maximum likelihood estimates of the parameters of each model, and selected the ‘best’ model based on the corresponding AIC values.

In order to overcome the issues raised by AIC discussed above, we decided to re-analyse the datasets and re-assess the models within a Bayesian framework.

## Methods

What follows is an elaboration of the description of nested sampling given by Skilling [6], [7].

### Nested sampling

The expected value of a function 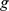 of a random variable 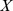 is given by
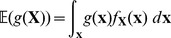
where 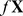 is the pdf of 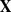. On comparing this expression with the target integral 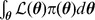, it is clear that
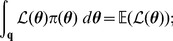
(5)that is to say, the expected value of the likelihood under the prior. The cumulative distribution function 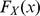 with respect to a random variable 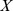 is defined by
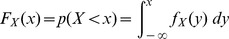
and is related to the expectation 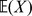 by
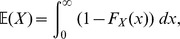

[25]; consequently, from (5), we obtain the important relationship
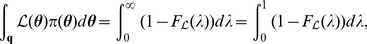
(6)where 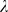 is likelihood, and 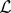 in the right-hand integral is equal to 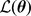. The reason why (6) is important is that the multivariate integral on the left-hand side has been equated to a univariate integral.

Since 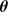 has a distribution defined by prior 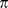, and 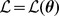, it follows that 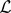 has a probability distribution and thus a cumulative distribution function,
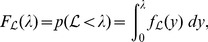
(7)which is present in the integrand of the right-hand integral of (6).

We can replace 
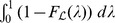
 in (6) with a more accessible integral by the following steps. First, since the pdf of 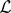 is connected to the pdf of 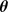 via 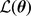, we can write
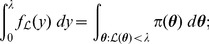
(8)thus, from (6), (7) and (8), we can write
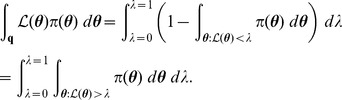
(9)


It will be convenient to rewrite the inner integral of (9) as 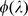 to give
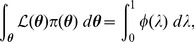
(10)where 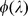 is the probability of selecting 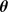 from the prior 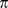 such that 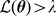:
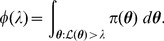
(11)


Introducing 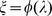, hence 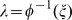, we can rewrite the previous integral as
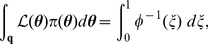
(12)where 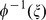 is that likelihood 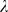 such that 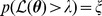 (cf. Equation (11)); for example, if 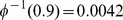 then 90% of 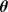 drawn from the prior 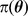 will have likelihoods greater than 0.0042.

### The algorithm

The main steps of the nested sampling technique are as follows. First, 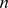 points 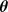 (i.e., parameter vectors) are sampled from the prior 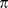, and their corresponding likelihoods 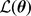 determined. The point 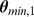 having the smallest likelihood is determined and its likelihood 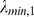 is recorded. Furthermore, the probability 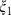 that 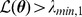 is also recorded.Point 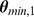 is replaced by a new 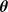 drawn from the prior 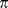 but restricted to those 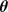 for which 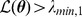. In other words, a *restricted prior* is used: 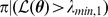. If 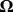 is the set of all possible 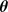 then the set 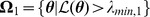 is a subset of 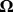.

The above sequence of determining 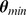 and the corresponding 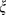 is performed on the new set of points, giving rise to 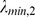 and 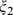. Point 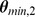 is replaced by a 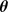 drawn from the new restricted prior 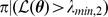. In other words, 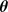 is sampled from 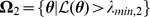, for which 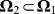.

This cycle is repeated until some stopping criterion has been reached. If this termination occurs at the 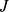-th iteration then the resulting values of 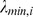 and 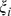 will be
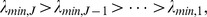


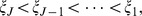
and the resulting sequence of 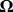 subsets is
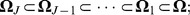
hence the term *nested sampling*.

**Figure 1:**
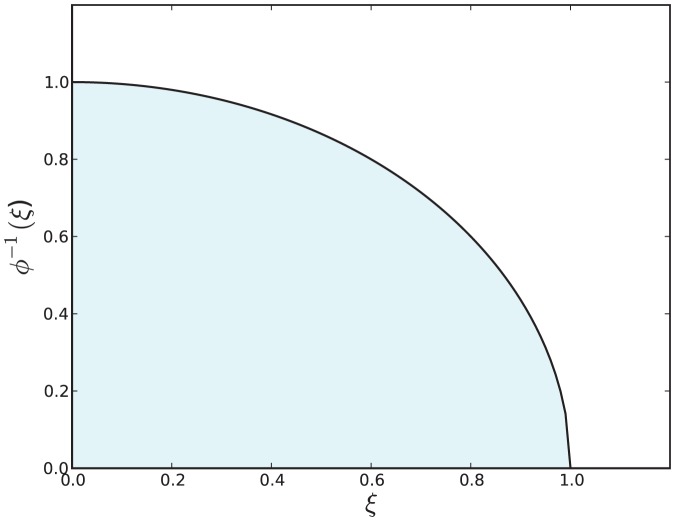
The shaded area below the curve for 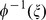 is equal to 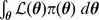. See Equation (12).

Model evidence 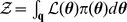 can be estimated from the recorded 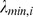 and 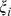 values by means of the approximation
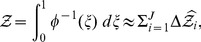
(13)where 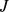 is the number of iterations used, and 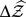 is a vertical rectangular segment under the curve of Figure 1.

Algorithm 1 (Table 1) describes the above process in pseudocode.

**Table 1:** Algorithm 1: The nested sampling algorithm.

**Input:** (a) likelihood function 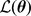 ; (b) prior 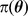 ; (c) number 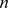 of active parameter vectors in use during nested sampling.
**Out put:** an estimate 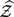 of 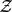 .
1: Let 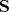 be a set of 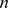 parameter vectors 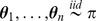
2: 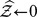
3: 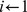
4:**while** terminating condition not satisfied **do**
5: 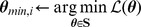
6: 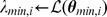
7: 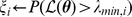
**8: if** 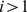 **then**
9: 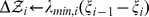 ▹Estimated segment of 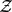
10: 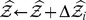
11: 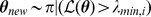 ▹Restricted prior
12: 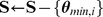
13: 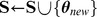
**return** 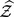

### Practical adjustments to the algorithm

We will now consider how some of the aspects of Algorithm 1 can be implemented.

Segment 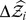 used in (13) could be evaluated by the trapezoidal approach
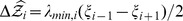
but Sivia and Skilling [26] have found
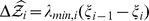
to be adequate (line 9 in Algorithm 1).

Line 7 in Algorithm 1 used the assignment 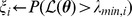, but an alternative approach is to replace this assignment with 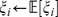. An approximation of 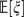 is derived as follows. Let 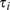 denote the ratio 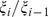, with 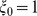. At the 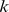
th iteration we have
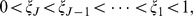
and so
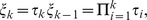
therefore,
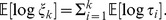
(14)Now,
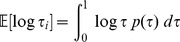


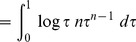

[27]

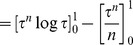


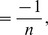
therefore, from (14),
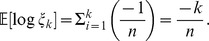



Since the logarithm function is strictly increasing and concave, we have, from Jensen's inequality, that
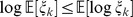
and thus
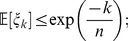
however, Sivia and Skilling [26, p. 186] drop the inequality and use the approximation
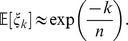



As regards the termination of Algorithm 1, there is no rigorous criterion as to when the algorithm should be stopped, but Skilling [7] and Feroz and Hobson [28] have found
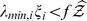
to be an effective stopping condition, where 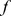 is the fraction of 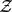 that will not significantly contribute to the estimate of 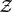 (according to a user-defined value).

Chopin and Robert [29] have shown that the asymptotic variance of the nested sampling approximation typically grows linearly with parameter dimensions.

Finally, there is the structure of the restricted priors. Each new point 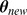 for a set 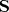 of active points is sampled from prior 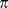 conditioned on the restriction that 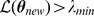. Rather than searching across the entire 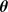 -space for such a point, it is more computationally efficient to restrict the search to a region 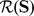 that contains 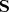. We have used rectangular cuboids for 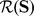.

Incorporating the above points into Algorithm 1 leads to Algorithm 2 (Table 2). Before applying the algorithm to our experimental datasets, we tested it on a simple two-parameter likelihood function 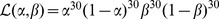. The analyses and results are presented in Methods S1.

**Table 2:** Algorithm 2: An implementation of Algorithm 1 in which practical adjustments are included.

**Input** (a) likelihood function 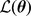 ; (b) prior 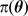 ; (c) number 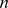 of active parameter vectors in use during nested sampling; (d) procedure for determining a region 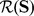 of parameter space that encloses a set of parameter vectors 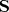 ; (e) fraction 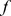 of 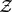 to be estimated.
**Output:** an estimate 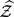 of 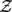 .
1: Let 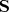 be a set of 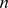 parameter vectors 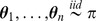
2: 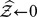
3: 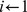
4:Repeat
5: 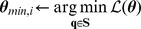
6: 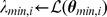
7: 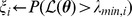
**8: if** 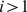 **then**
9: 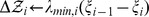 ▹Estimated segment of 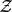
10: 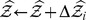
11: 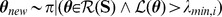 ▹Restricted prior
12: 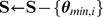
13: 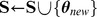
14: **until:** 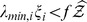 ▹The stopping condition
**return** 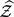

### The *Salmonella* models

Evidence 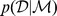 was estimated by nested sampling with respect to two groups of models associated with within-host *S. enterica* infection, were each model 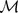 provides an expression for the probability 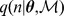 that a host cell contains 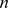 bacteria.

In the first group of models, infected cells are assumed to burst when the number of bacteria they contain reach a fixed threshold 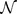. The probability distributions considered for 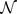 are shown in Table 3.

**Table 3:** Probability distributions 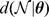 for the burst thresholds 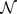.

Model	Distribution	Parameters, *θ*
1	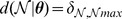	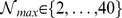
2	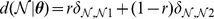	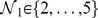 , 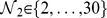 , 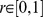
3	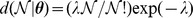	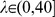
4	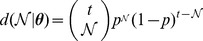	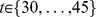 , 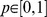
5	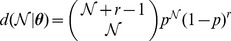	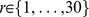 , 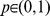
6	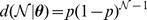	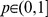

(1) Unimodal Kronecker, (2) bimodal Kronecker, (3) Poisson, (4) binomial, (5) negative binomial, and (6) geometric.

For the second group of models, the assumption is that, instead of pre-programmed burst thresholds 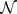, there is burst rate 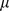 that is a function of the number of bacteria 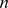 in a cell. For these models, the general relationship is
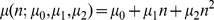
(15)where 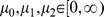. Furthermore, the rate of bacterial replication 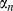 is assumed to be related to 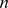 by
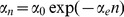
(16)where 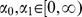. As explained in Brown et al. [24], in the dynamic model, time can be re-scaled by the baseline replication rate 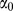, therefore this parameter cannot be estimated using the quasi-stationary distribution. For convenience, we set 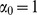, so that the values of other parameters are relative to the baseline replication rate. The parameters of the eight stochastic models considered are shown in Table 4.

**Table 4:** Parameters used for the eight stochastic models based on (15) and (16).

	Parameters, *θ*				
Model	*μ*_0_	*μ*_1_	*μ*_2_	*α*_0_	*α_e_*
7	*μ*_0_	0	0	1	0
8	0	*μ*_1_	0	1	0
9	0	0	*μ*_0_	1	0
10	*μ*_0_	*μ*_1_	*μ*_2_	1	0
11	*μ*_0_	0	0	1	*α_e_*
12	0	*μ*_1_	0	1	*α_e_*
13	0	0	*μ*_2_	1	*α_e_*
14	*μ*_0_	*μ*_1_	*μ*_2_	1	*α_e_*

For each model, some of the parameters were set equal to constant values, which effectively removed the parameters from the model. The range of values considered were 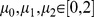 and 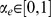.

Under the assumption that the number of host cells infected by 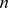 bacteria reaches a quasi-stationary distribution, the probability 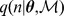 that a cell contains 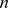 bacteria can be derived for the 14 models [30]. For Model 1, we have the relationship
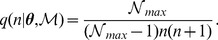
(17)For Models 2 to 6, the relationship is
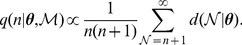
(18)For Models 7 to 16, we have the recursive relationship
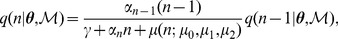
(19)where the infection rate constant 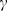 is given by
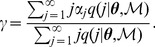
(20)


The value for q(1| 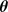, 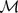) can be handled as follows. Let
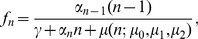
(21)so that (19) can be written as 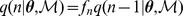, then
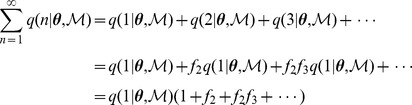
but 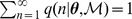; therefore,
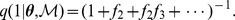



When bacterial replication is not dependent on 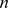, 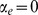, in which case 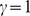, but when replication is density dependent, (19) and (20) need to be solved self-consistently. This can be done by assuming an initial value for 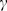, computing 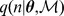 from (19), updating 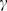 using (20), and repeating this iteratively until 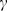 no longer changes significantly. This process is shown in Algorithm 3 (Table 5).

**Table 5:** Algorithm 3: Estimation of 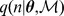 using an iterative estimation of the infection rate constant 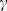.

**Input:** parameters 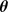 for model 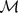 .
**Output:** an estimate of probabilities 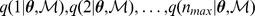 .
1: 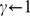 Initial value for 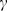
2: 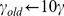
3: **while:** 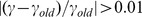 **do**
4: 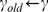
5: 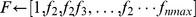
where 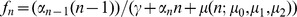 ▹Equation (21)
6: 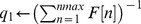 ▹Estimate of 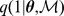
7: 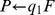 ▹Estimates of 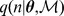 where 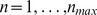
8: 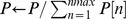 ▹Normalisation of the estimated probabilities
9: 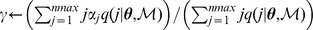
**return:** 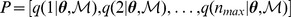

### Likelihood function

With expressions for 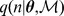 established for all the models, we can now determine the likelihood 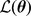 required for Algorithm 2. Following Brown et al. [30], we can express the likelihood function by a multinomial distribution:
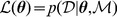
(22)

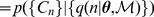
(23)

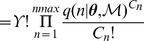
(24)where 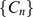 is the observed distribution of 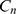 (the number of cells with 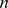 bacteria), and 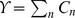, if observations are assumed to be independent. Garca-Pérez [31] provides an algorithm for the accurate computation of multinomial probabilities.

As regards the prior 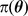 for a model 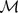, it will be assumed to be uniform across the parameter space of interest for that model; consequently, the prior will be set equal to the reciprocal of the size of the parameter space. More precisely,
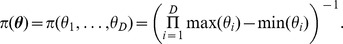



### A continuation approach

The theory underlying nested sampling assumes that all the parameters for a model have continuous values, however, this will not necessarily be the case in practice. For example, the binomial model (Model 3) has a discrete parameter 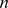 and a continuous parameter 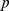.

It is possible to formulate a theory of nested sampling for discrete parameters by replacing integrals with summations, but modifications to Algorithm 2 would be required to take account of the fact that, if 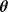 is discrete, several points could occupy the same location in parameter space.

An alternative response to the presence of discrete parameters is to use a type of *continuation approach* [32]; in other words, if 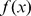 is a function defined only for integer values of 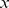, replace it with another function 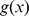 that takes real values, but for which 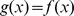 when 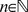 (or 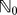).

For Model 2, the Kronecker delta 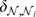 can be replaced with a narrow Gaussian function 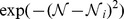 with 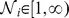. In the case of Model 1, continuation can be applied directly to (17) by allowing 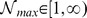.

For those models using a factorial of a parameter (i.e., Models 4 and 5), we can replace 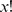 with 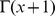 since 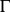 is a function of a real value.

### The data

The data 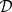 consisted of the number 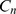 of mice cells observed (via fluorescence microscopy) to contain 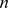 *S. enterica* bacteria: 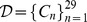. One dataset was used for a virulent bacterial strain (SL5560); another for an attenuated strain (SL3261). The infected cells were taken randomly from various locations in the liver. The observed 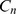 values are shown in Table 6.

**Table 6:** The number *C_n_* of cells containing *n* bacteria when virulent (SL5560) and attenuated (SL3261) strains of bacteria were used.

	*C_n_*	
*n*	Virulent	Attenuated
1	655	1189
2	250	396
3	87	104
4	86	70
5	54	40
6	42	25
7	13	8
8	30	10
9	8	9
10	19	3
11	5	7
12	12	4
13	5	3
14	1	4
15	6	0
16	3	2
17	2	1
18	0	2
19	1	1
20	4	0
21	0	0
22	0	0
23	0	0
24	0	1
25	1	0
26	0	0
27	0	0
28	0	0
29	1	0

The data was pooled. If 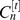 denotes the number of cells having 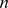 bacteria on day 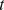 then, for the virulent strain, Brown et al. [24] used 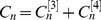, and for the attenuated strain they used 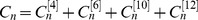.

### Posterior model probabilities

If we assume that the set of candidate models is exhaustive, we can apply (2) to estimate the posterior probability 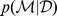 for each model. Furthermore, if 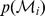 is assumed to be equal for all models, we can use
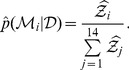
(25)


**Figure 2:**
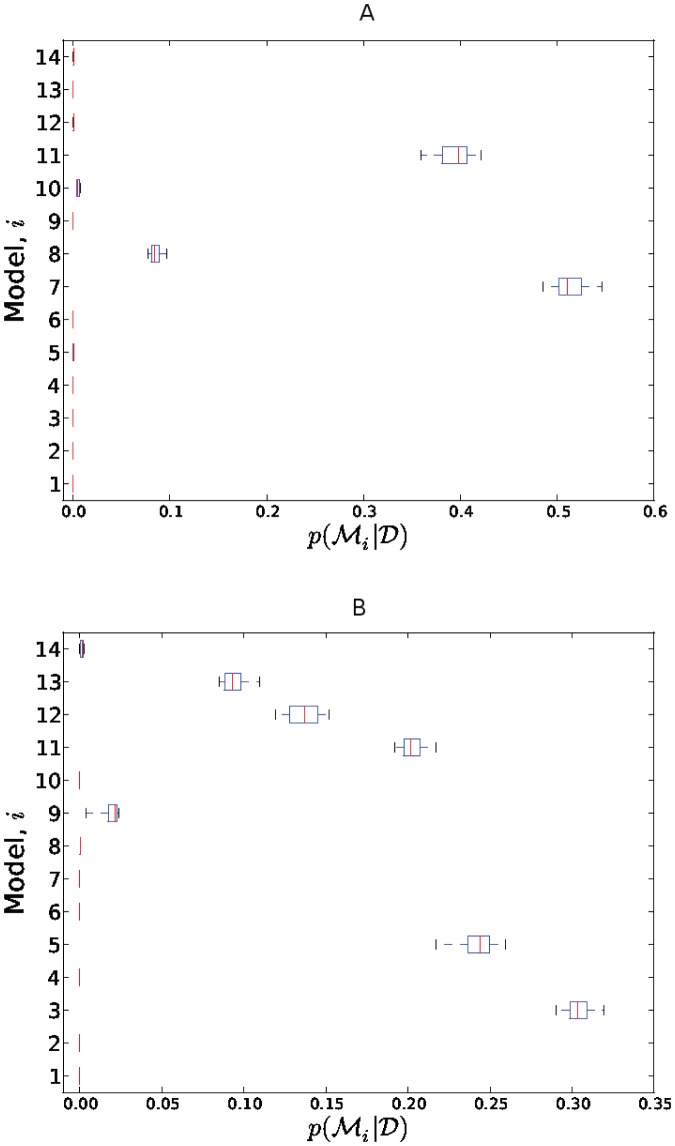
Estimates of the posterior model probabilities 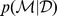 when using data from (A) the attenuated strain and (B) the virulent strain.

There are 14 models, each arbitrarily having 10 estimates of 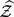, but it is impractical to systematically apply each of the 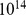 possible combinations of 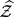 to [25]; therefore, the 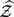 values were chosen randomly in order to obtain distributions for 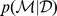. The resulting distributions are shown in Figure 2.

An alternative approach to Bayesian model comparison is to use the Bayes factor 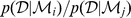. This provides a relative comparison of models 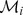 and 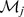 but not the absolute values of their posterior probabilities 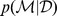.

## Results

The estimated model-evidence values 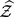 obtained by nested sampling for each model is shown in Tables 7 and 8. The ranges are shown in Table 9.

**Table 7:** Median 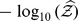 estimated for Models 1 to 6.

Model	Distribution	Attenuated	Virulent
1	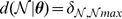	77.59	38.56
2	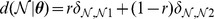	69.49	92.79
3	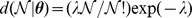	53.75	34.09
4	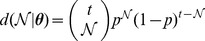	245.87	281.46
5	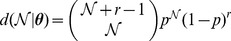	30.26	34.18
6	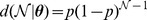	84.26	79.97

The highest model evidence 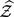 (bold) and second highest model evidence (italic) models are highlighted.

**Table 8:** Median 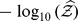 estimated for stochastic Models 7 to 14.

	Parameters, *θ*						
Model	*μ*_0_	*μ*_1_	*μ*_2_	*α*_0_	*α_e_*	Attenuated	Virulent
7	*μ*_0_	0	0	1	0	27.21	38.56
8	0	*μ*_1_	0	1	0	28.00	36.93
9	0	0	*μ*_2_	1	0	38.80	35.24
10	*μ*_0_	*μ*_1_	*μ*_2_	1	0	29.21	39.21
11	*μ*_0_	0	0	1	*α_e_*	27.32	34.27
12	0	*μ*_1_	0	1	*α_e_*	30.13	34.43
13	0	0	*μ*_2_	1	*α_e_*	41.25	34.60
14	*μ*_0_	*μ*_1_	*μ*_2_	1	*α_e_*	30.04	36.34

The highest model evidence 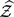 (bold) and second highest model evidence (italic) models are highlighted.

**Table 9:** 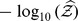
 estimates for all models.

	Attenuated			Virulent		
Model	min	median	max	min	median	max
1	77.56	77.59	77.63	38.55	38.56	38.58
2	69.38	69.49	69.66	92.66	92.79	92.88
3	53.71	53.75	53.79	34.07	34.09	34.10
4	245.83	245.87	245.91	281.36	281.46	281.50
5	29.93	30.26	30.52	34.16	34.18	34.24
6	84.23	84.26	84.30	79.93	79.97	80.01
7	27.19	27.21	27.24	38.52	38.56	38.58
8	27.94	28.00	28.02	36.88	36.93	36.97
9	38.78	38.80	38.85	35.20	35.24	35.98
10	29.06	29.21	29.39	38.66	39.21	43.12
11	27.28	27.32	27.38	34.24	34.27	34.28
12	29.99	30.13	30.36	34.39	34.43	34.50
13	40.93	41.25	41.84	34.53	34.60	34.63
14	29.86	30.04	30.48	36.19	36.34	39.65

With respect to the data from the attenuated strain, the most probable model was Model 7 (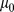 only) followed by Model 11 (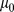 and 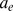). With respect to the data from the virulent strain, the most probable model was Model 3 (Poisson) followed by Model 5 (negative binomial).

### Parameter distributions

After having estimated the most probable model, 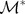, it is of interest to estimate the posterior joint probability of the parameters 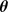 with respect to 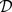 and 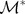: 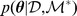.

From Bayes' theorem, we can write
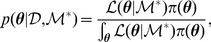
(26)and the denominator of Eqn (26) can be estimated by nested sampling:
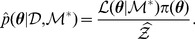
(27)


### Parameter estimation via reject sampling

Distribution 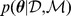 can be estimated using reject sampling with approximation (27). As part of this process, the maximum of 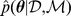 can be determined by performing Nelder-Mead simplex optimisation with respect to this distribution over parameter space.

**Figure 3:**
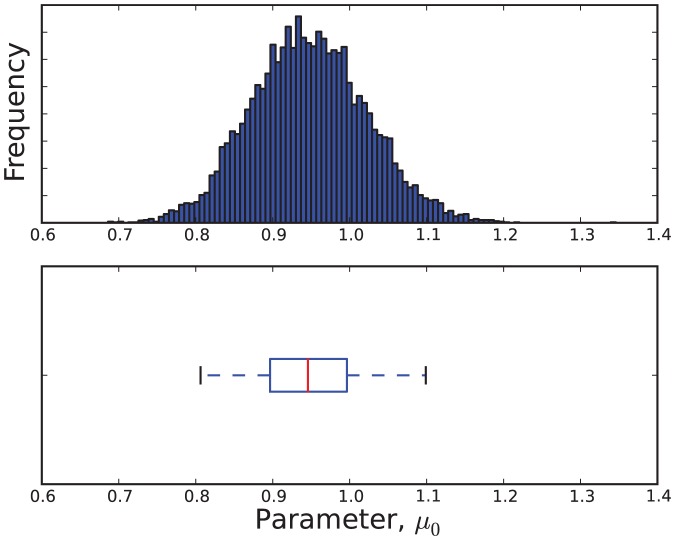
An estimate of the marginal probability distribution 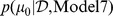. 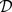
 is data from the attenuated strain.

**Figure 4:**
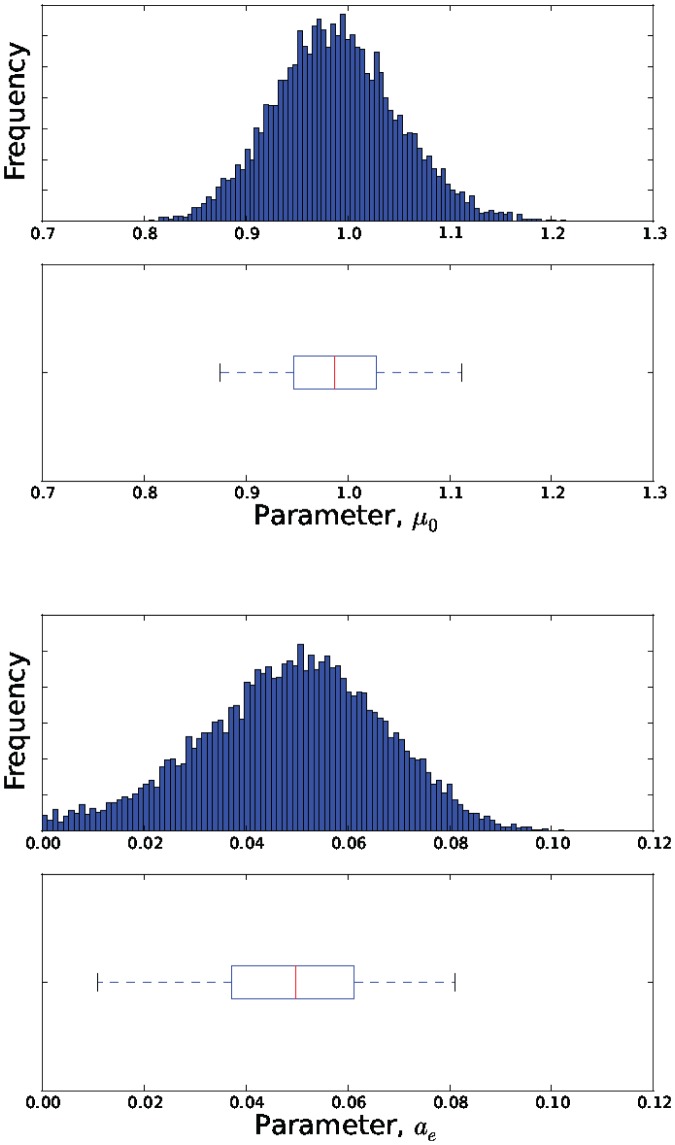
Estimates of the marginal probability distributions 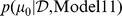 and 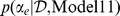. 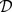
 is data from the attenuated strain.

**Figure 5:**
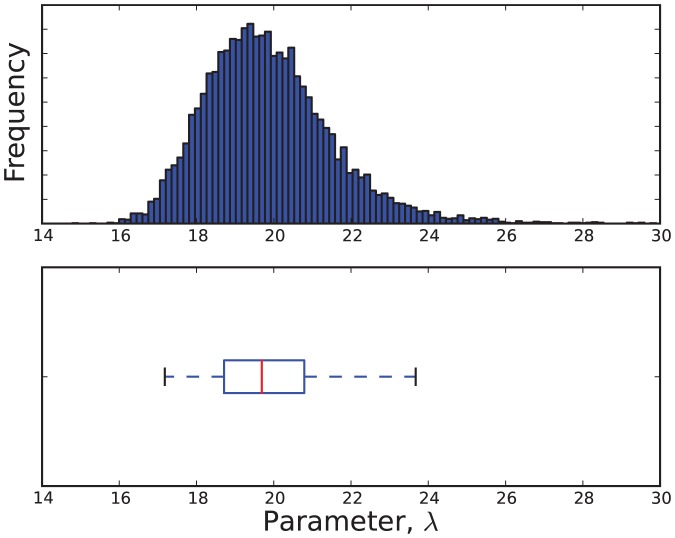
An estimate of the marginal probability distribution 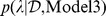. 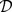
 is data from the virulent strain.

**Figure 6:**
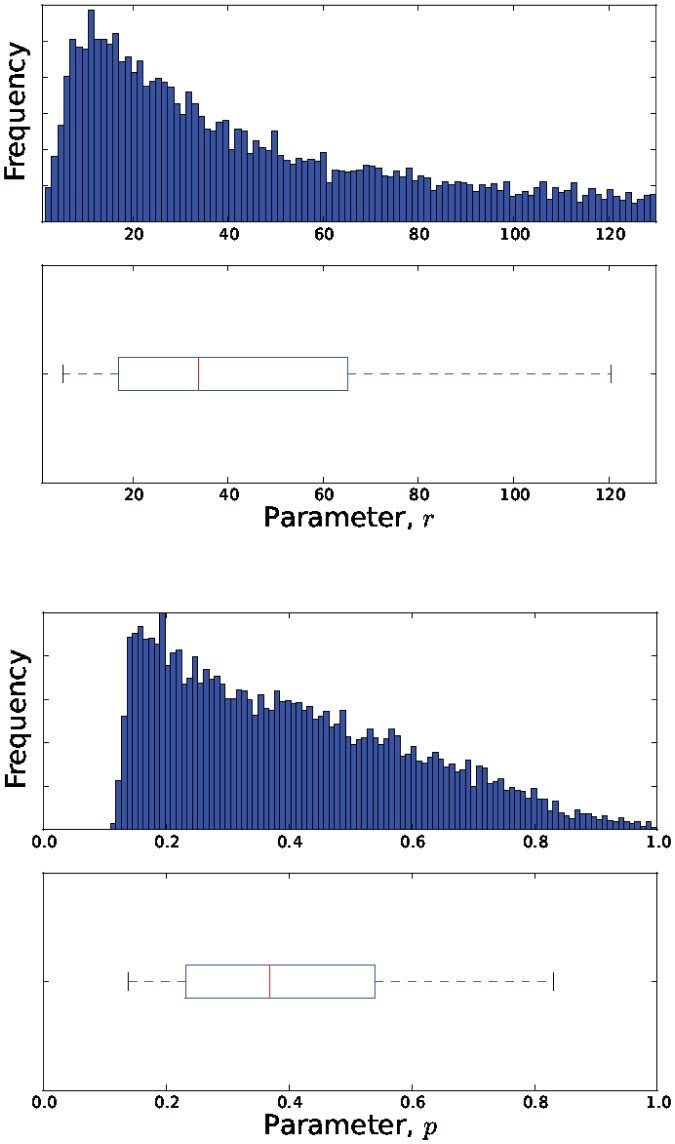
Estimates of the marginal probability distributions 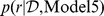 and 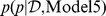. 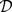
 is data from the virulent strain.

**Figure 7:**
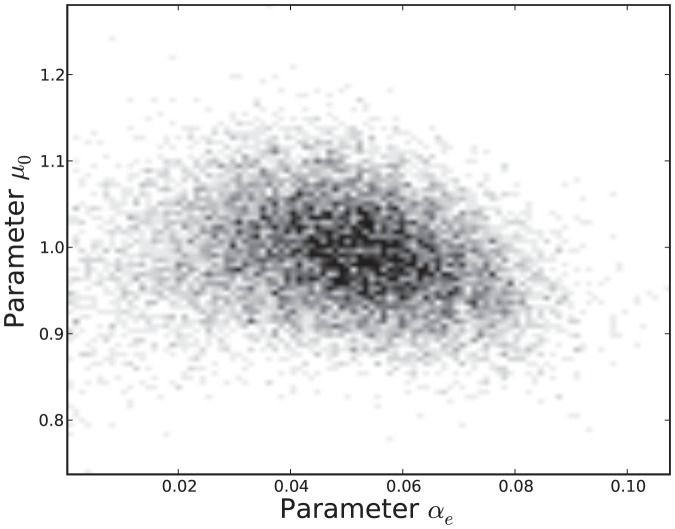
Density scatter plot of the estimated joint probability distribution 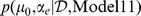. 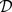
 is data from the attenuated strain.

**Figure 8:**
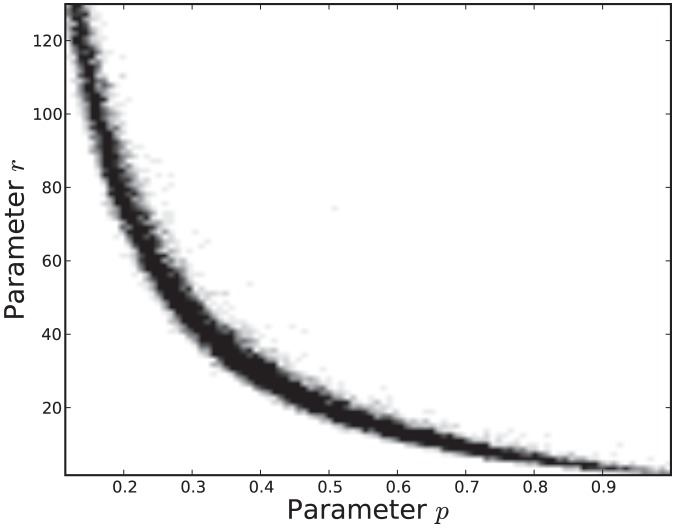
Density scatter plot of the estimated joint probability distribution 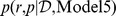. 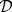
 is data from the virulent strain.

The estimated parameter distributions obtained by reject sampling for Models 3, 5, 7 and 11, are shown in Figures 3, 4, 5, and 6, respectively. In each case, the sample size 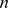 was 10000. The samples obtained by reject sampling were also used to construct density scatter plots (Figures 7 and 8), which provide a visualisation of the correlations between the parameters.

### Parameter estimation directly from nested sampling

The parameter sequence 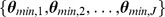 is produced during nested sampling. Can this set of parameters be regarded as a random sample from 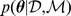
? Sivia and Skilling [26] proposed using 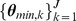 for this purpose so long as it is weighted by 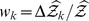, where 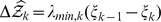, on the basis that 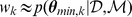. A theoretical justification for this is given by Chopin and Robert [29].

The appropriateness of regarding 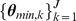 as a random sample from 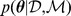, was ascertained empirically using the Kolomogorov-Smirnov test, as follows.

The Kolmogorov-Smirnov statistic 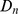 is given by
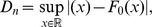
where 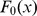 is the cdf of the null-hypothesis pdf, and 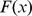 is the empirical cdf obtained from a sample 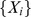:
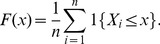
(28)This definition can be generalized to a weighted Kolmogorov-Smirnov statistic by replacing (28) with a weighted cdf:
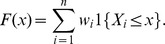
This allows us to take account of the weights 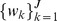 on 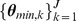.

Applying this method to the toy model 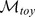 presented in Methods S1, a sample 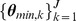, with 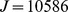, was obtained by performing nested sampling for the evaluation of evidence 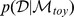, where 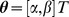. The corresponding sample 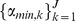 was compared with the marginal beta distribution,
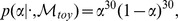
using the weighted Kolmogorov-Smirnov statistic 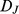. This statistic was equal to 0.01298. In order to obtain a frequentist 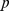-value for the statistic, an empirical probability distribution for 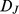 was obtained by randomly selecting a set 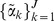 of 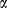 values from 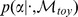 and determining 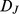 for the set, this being done 10000 times. On comparing 0.01298 with this empirical distribution, the 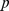-value for 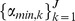 was found to be 0.0276. In contrast, when a sample of size 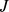 was obtained by reject sampling from 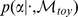, the value of unweighted 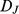 was 0.00630, which has a 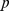-value of 0.5772.

As a result of this experiment, it was decided not to use 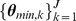 for estimating parameter distributions.

### Model checking

It does not follow that the most probable model from a set of candidate models is necessarily an acceptable model: the most probable model may be the least worst of a set of poor models. What is required is an assessment of the fit of the most probable models to the observed data.

A common approach to assessing the fit of a model to data is to use a 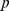-value with respect to some statistic 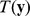, where 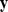 is observed data. More formally, the classical 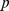-value is given by
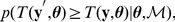
(29)where 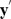 is a possible future value, and the probability is taken over the distribution of 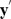 given 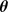, a single parameter estimate.

A drawback of (29) is that it does not take account of the uncertainty in 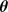 expressed by the posterior distribution 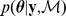. In contrast, the Bayesian *posterior predictive* 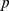 *-value* [33], [34]

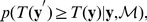
(30)overcomes the problem by using the *posterior predictive distribution*:
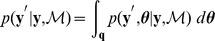


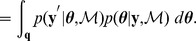



The posterior distribution can be simulated by drawing 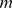 values 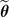 from 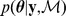, and then, for each 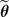, sampling a 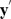 from 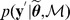. The resulting 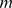 values of 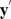 represent draws from 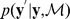.

In the context of the *Salmonella* study, 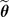 was provided by the parameter estimates obtained for 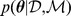, 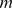 was set to 10000, and 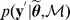 was modelled as a multinomial distribution
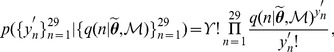
(31)where 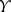 is the total number of counts (cf. (22)).

In order to obtain 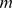 values of 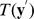 drawn from 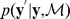, each 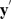 drawn from 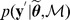 is mapped to 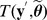.

We used the 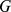-statistic for the test statistic 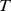 [35]. The 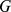-statistic is proportional to the Kullback-Leibler measure of distribution divergence, and is given by
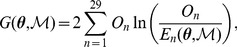
(32)where 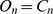, and 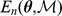 is the expected value for 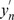: 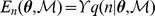.

Applying the above approach for estimating the distribution of 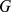 under a given model 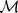, the posterior predictive 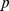-values for 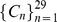 were found to be 0.005 for Model 7 and 0.006 for Model 11 (with respect to the attenuated strain), 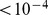 for Model 3 and 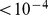 for Model 5 (with respect to the virulent strain). This suggests a poor fit of the models to the data.

**Figure 9:**
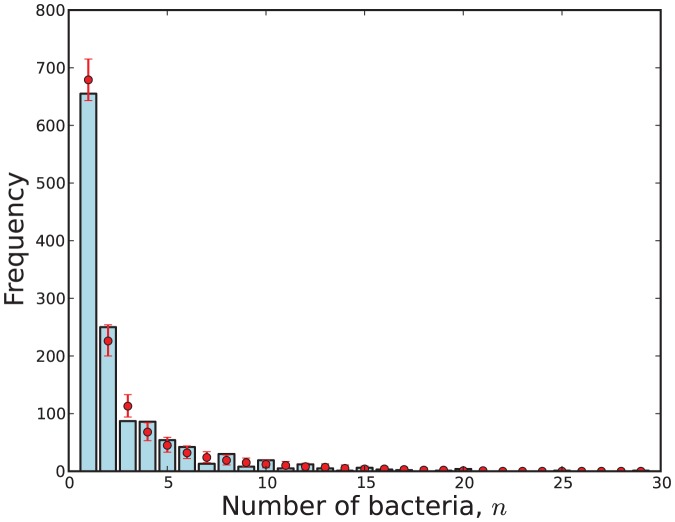
The observed number of cells with 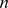 bacteria (blue) compared with 95% credibility intervals (red) predicted by Model 3 with respect to the virulent strain.

**Figure 10:**
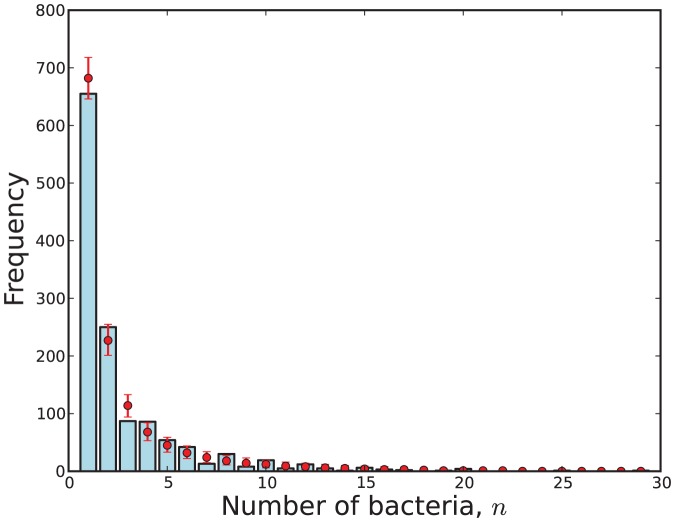
The observed number of cells with 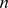 bacteria (blue) compared with 95% credibility intervals (red) predicted by Model 5 with respect to the virulent strain.

**Figure 11:**
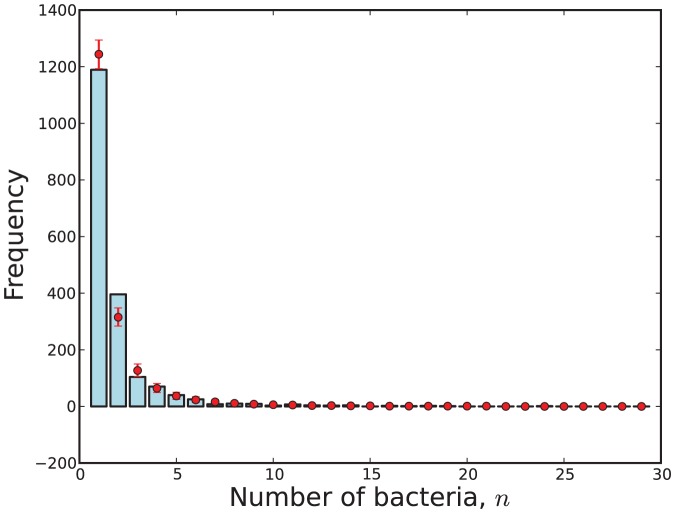
The observed number of cells with 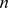 bacteria (blue) compared with 95% credibility intervals (red) predicted by Model 7 with respect to the attenuated strain.

**Figure 12:**
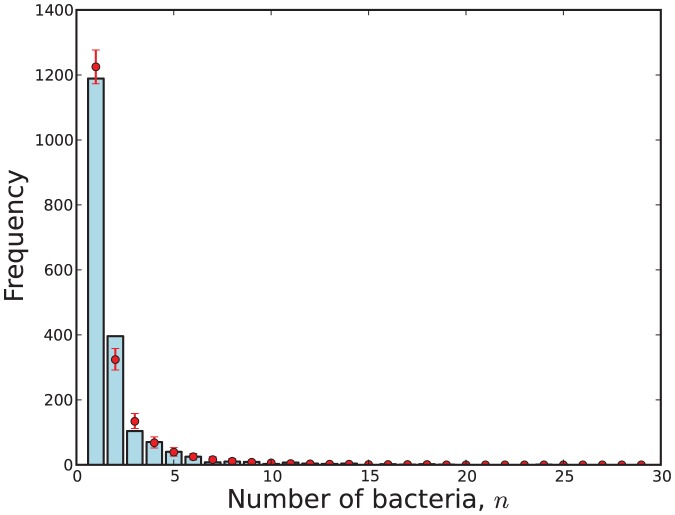
The observed number of cells with 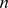 bacteria (blue) compared with 95% credibility intervals (red) predicted by Model 11 with respect to the attenuated strain.

A visual representation of the fit of data to a model 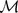 can be provided by comparing the observed count 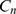 (the number of cells containing 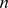 bacteria) to the distribution of 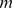 possible count values 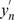 obtained via (31). This visualisation is shown in Figures 9, 10, 11 and 12.

## Discussion

The AIC is a common maximum-likelihood approach to model comparison, but nested sampling enables a Bayesian approximation of model evidence 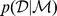 to be computed, along with the advantages of adopting the Bayesian approach. These include integration across parameters; estimation of the posterior parameter distributions (with visualisation of parameter correlations); and estimation of the posterior predictive distributions for goodness-of-fit assessments of the models.

Under the assumptions used, the most probable models with respect to the virulent and attenuated strains of *S. enterica* were burst-threshold Model 3 (Poisson) and burst-rate Model 7 (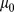 only), respectively. The next two most probable models were burst-threshold Model 5 (negative binomial) and burst-rate Model 11 (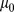 plus 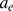), respectively. However, the Bayesian posterior predictive 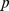-values indicate that alternative models and/or a relaxation of the quasi-stationary assumption adopted by Brown et al. [24] should be considered. It may be the case that one of the candidate models is correct but the use of pooled data was detrimental.

Other assumptions of the underlying mechanistic model may also be wrong; in particular, the absence of bacterial death and the assumption that each released bacterium infects a new macrophage.

For both the attenuated and virulent strains, the data 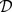 was recorded over a number of days following infection and then pooled, with 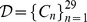. If time-dependent data is to be retained and nested sampling is to be applied then a method is required to estimate the likelihood function 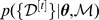, where 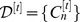 and 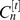 is the number of cells containing 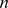 bacteria on the 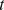-th day. Branching processes have been used to model a variety of biological systems [36], and we will investigate the potential of estimating 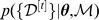 through the use of Bellman-Harris processes to model within-host infection dynamics.

We have demonstrated that a visualisation of the marginal and joint posterior parameter distributions 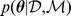 is readily obtainable once model evidence 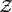 has been estimated by nested sampling. The estimated joint posterior distributions provided a visualisation of the correlations between the parameters. Through the use of a weighted Kolomogorov-Smirnov test, we also found that the parameter sequence 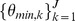 resulting from nested sampling could not be regarded as a random sample from the posterior parameter distribution 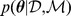.

One drawback of Algorithm 2 is that the restricted priors will converge to a single mode when a likelihood is multi-modal, and this will cause the evidence 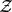 to be underestimated. This issue can be resolved by implementing a multi-modal version of nested sampling, such as that proposed by Feroz et al. [37] for comparing cosmological models.

## Supporting Information

Methods S1 **Toy example of nested sampling.**
(PDF)
