# Supplementary material for: Nested Sampling for Bayesian Model Comparison in the Context of Salmonella Disease Dynamics
Source: PLoS One. 2013 Dec 20;8(12):e82317. doi: 10.1371/journal.pone.0082317 (PMC3869703; doi:10.1371/journal.pone.0082317)
Supplement: Methods S1 — Toy example of nested sampling. (PDF) [file pone.0082317.s001.pdf]

Supplementary Methods S1
Nested sampling for Bayesian model comparison in the context of Salmonella disease dynamics

Richard Dybowski, *et al*

1 Toy example of nested sampling

Consider $\mathcal{Z} = \int_{\boldsymbol{\theta}} \mathcal{L}(\boldsymbol{\theta})\pi(\boldsymbol{\theta})d\boldsymbol{\theta}$ with $\boldsymbol{\theta} = [\alpha, \beta]^{\mathsf{T}}$ and $\mathcal{L}(\alpha, \beta) = \alpha^{30}(1 - \alpha)^{30}\beta^{30}(1 - \beta)^{30}$ (See Figure S1). If $\pi(\boldsymbol{\theta})$ is a uniform prior over the domain $[0, 1]^2$ then $\pi(\boldsymbol{\theta}) = 1$, and

$$
\begin{aligned}
\mathcal{Z} &= \int_{\alpha=0}^{\alpha=1} \int_{\beta=0}^{\beta=1} \mathcal{L}(\alpha, \beta)\; d\alpha\; d\beta \\
&= \int_0^1 \alpha^{30}(1-\alpha)^{30}\; d\alpha \int_0^1 \beta^{30}(1-\beta)^{30}\; d\beta \\
&= \left(\frac{\Gamma(31)\Gamma(31)}{\Gamma(62)}\right)^2 \\
&\approx 1.9215 \times 10^{-38}\;.
\end{aligned}
$$

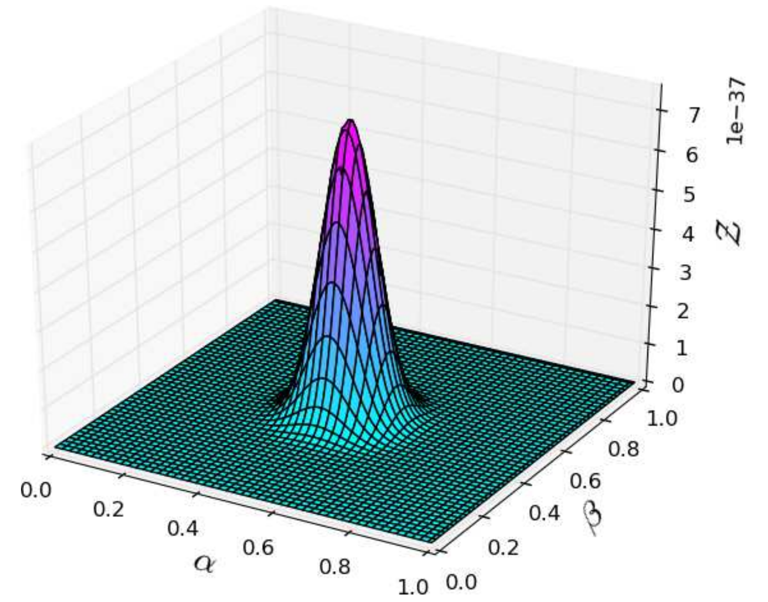

Figure S 1: Hypothetical $\mathcal{Z}$ where $\mathcal{L}(\alpha, \beta) = \alpha^{30}(1 - \alpha)^{30}\beta^{30}(1 - \beta)^{30}$ and $\pi(\boldsymbol{\theta}) = 1$.

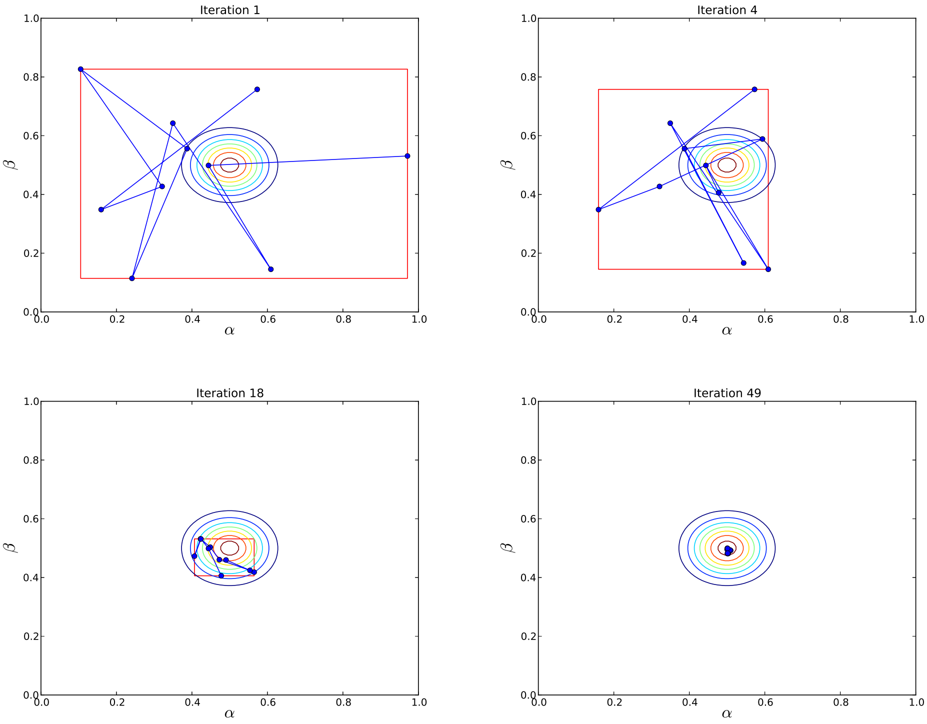

Figure S 2: Convergence of $n = 10$ points $\boldsymbol{\theta} = [\alpha, \beta]^{\mathsf{T}}$ when nested sampling was applied to the toy example of Section 1. The locations of the points (highlighted by lines between the points) are shown for the 1st, 4th, 18th and 49th iterations. The red rectangles are the boundaries of successive restricted-prior regions.

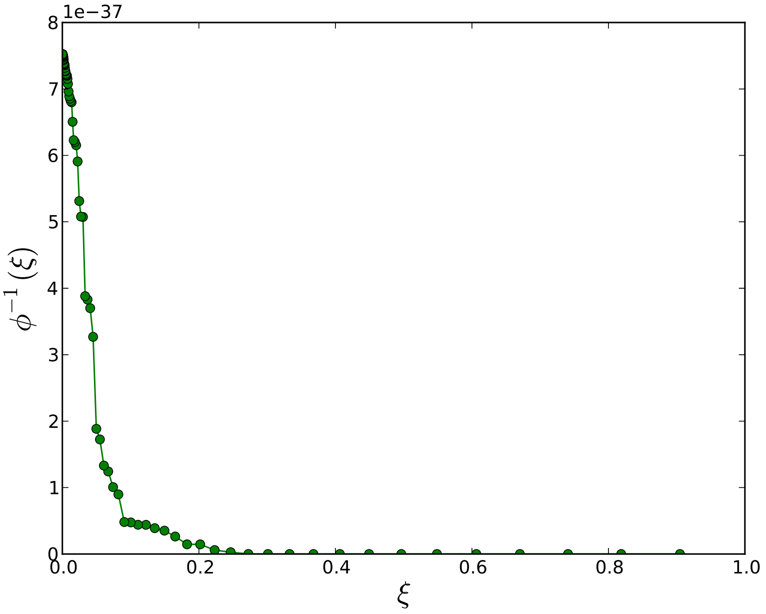

Figure S 3: Plot of $\phi^{-1}(\xi)$ vs. ξ obtained from the nested sampling visualised in Figure 2, where $\phi^{-1}(\xi) = \lambda_{min}$.

Figure S2 shows the convergence of $n = 10$ points $\boldsymbol{\theta}$ when nested sampling was applied to the toy example. Figure 3 shows the resulting plot of $\phi^{-1}(\xi)$ vs. ξ, where $\phi^{-1}(\xi) = \lambda_{min}$, from which $\widehat{\mathcal{Z}}$ is obtained numerically:

$$\widehat{\mathcal{Z}} = \sum_{i=1}^{J} \Delta \widehat{\mathcal{Z}}_i = \sum_{i=1}^{J} \lambda_{min,i}(\xi_{i-1} - \xi_i) \; .$$

The accuracy with which the nested sampling algorithm can estimate $\mathcal{Z}$ was tested using $n = 10$, 100 and 1000 active points. The constrained prior regions $\mathcal{R}(\mathbf{S})$ were rectangular (with enlargement factor δ set to 1), and $f = 0.01$ was used for the stopping condition. The algorithm was repeated 10 times for each value of n, and the results are shown in Table S1.

Table S 1: The effect of n on percentage error with respect to the toy model. The distributions of the percentage errors for each n are given in terms of the minimum, first quartile (Q_1), median, third quartile (Q_3) and maximum. The median number of iterations required, $\tilde{J}$, is also given. In each case, $f = 0.01$.

n	minimum	Q_1	median	Q_3	maximum	$\tilde{J}$
10	48	82	148.5	199	370	74
100	-22	3.4	26.0	33.5	41	805
1000	-8.6	-4.2	-1.6	3.4	5.1	8289

Table S 2: The effect of f on percentage error with respect to the toy model. In each case, $n = 1000$.

f	minimum	Q_1	median	Q_3	maximum	$\tilde{J}$
0.01	-8.6	-4.2	-1.6	3.4	5.1	8289
0.005	-10.3	-5.80	-0.94	1.25	6.1	8975.5
0.001	-3.2	2.1	2.2	5.4	11.7	10554

2 Parameter estimation

The estimated parameter distributions obtained by reject sampling for the toy example are shown in Figure S4. The resulting samples were also used to construct a density scatter plot (Figure S5).

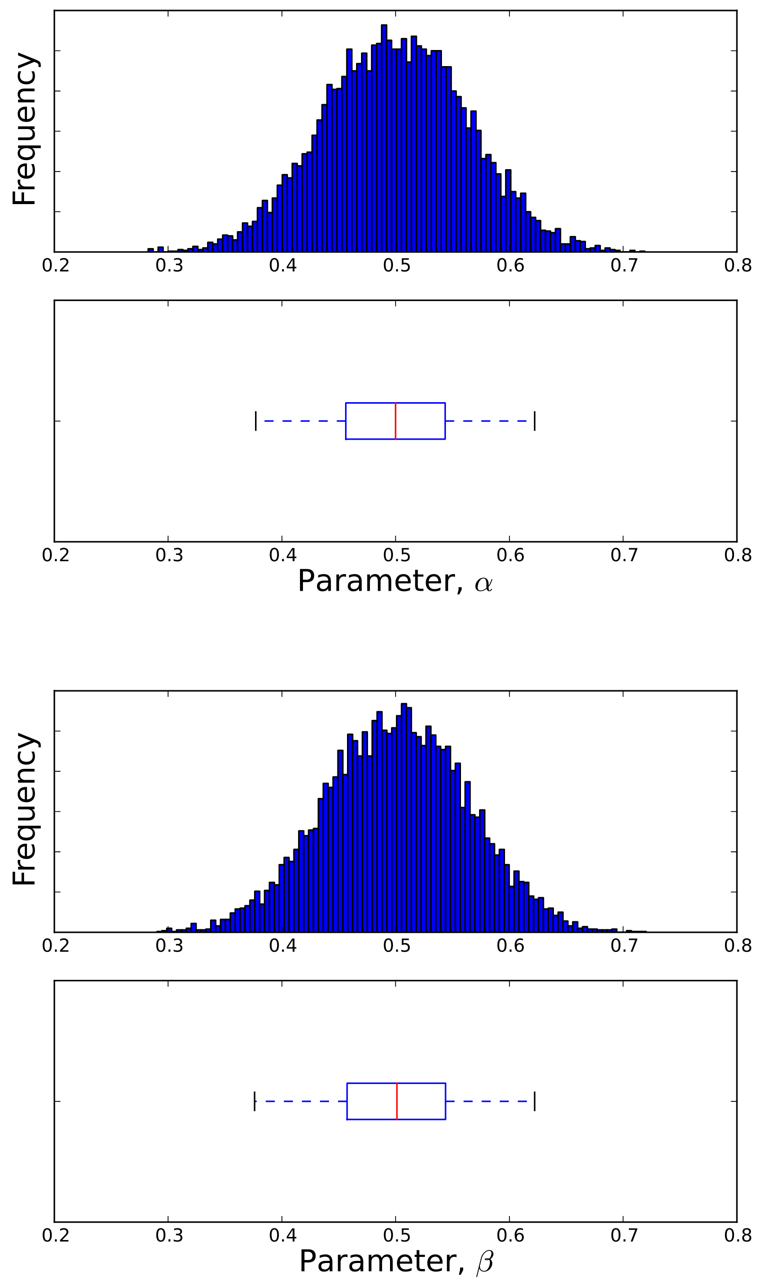

Figure S 4: Estimations of the marginal probability distributions $p(\alpha|\cdot, \mathcal{M}_{toy})$ and $p(\beta|\cdot, \mathcal{M}_{toy})$ based on the toy example given in Section 1. The ends of the whiskers correspond to the 2.5th and 97.5th percentiles, and thus define 95% credibility intervals.

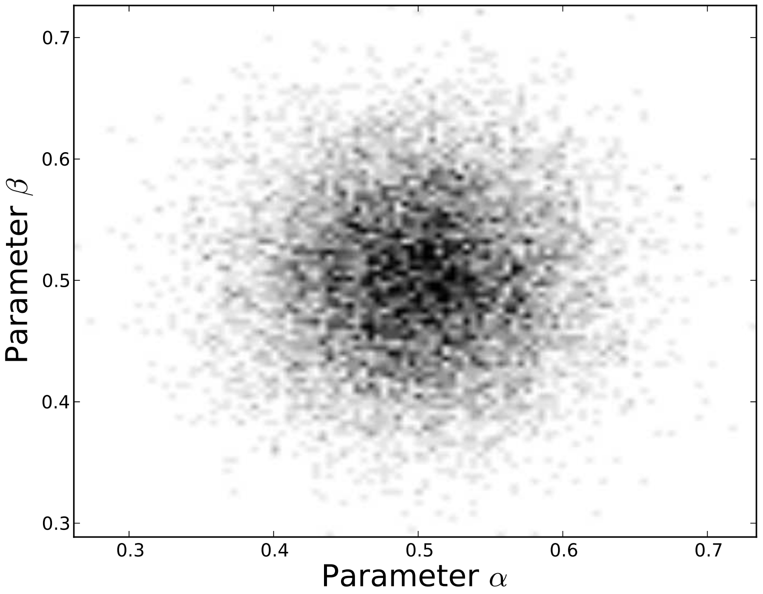

Figure S 5: Density scatter plot of the estimated joint probability distribution $p(\alpha, \beta|\cdot, \mathcal{M}_{toy})$.
